# Boronic Acid Transition State Inhibitors as Potent Inactivators of KPC and CTX-M β-Lactamases: Biochemical and Structural Analyses

**DOI:** 10.1128/aac.00930-22

**Published:** 2023-01-05

**Authors:** Tahani A. Alsenani, María Margarita Rodríguez, Barbara Ghiglione, Magdalena A. Taracila, Maria F. Mojica, Laura J. Rojas, Andrea M. Hujer, Gabriel Gutkind, Christopher R. Bethel, Philip N. Rather, Maria Luisa Introvigne, Fabio Prati, Emilia Caselli, Pablo Power, Focco van den Akker, Robert A. Bonomo

**Affiliations:** a Department of Biochemistry, Case Western Reserve University School of Medicine, Cleveland, Ohio, USA; b Universidad de Buenos Aires, Facultad de Farmacia y Bioquimica, Instituto de Investigaciones en Bacteriologia y Virologia Molecular, Buenos Aires, Argentina; c Consejo Nacional de Investigaciones Científicas y Tecnicas, Buenos Aires, Argentina; d Department of Medicine, Case Western Reserve University School of Medicine, Cleveland, Ohio, USA; e Louis Stokes Cleveland Department of Veterans Affairs Medical Center, Cleveland, Ohio, USA; f Department of Molecular Biology and Microbiology, Case Western Reserve University School of Medicine, Cleveland, Ohio, USA; g CWRU—Cleveland VAMC Center for Antimicrobial Resistance and Epidemiology, Cleveland, Ohio, USA; h Department of Microbiology and Immunology, Emory University, Atlanta, Georgia, USA; i Emory Antibiotic Resistance Center, Emory University, Atlanta, Georgia, USA; j Research Service, Atlanta VA Medical Center, Decatur, Georgia, USA; k Department of Life Sciences, University of Modena and Reggio Emilia, Modena, Italy; l Department of Pharmacology, Case Western Reserve University School of Medicine, Cleveland, Ohio, USA; m Department of Proteomics and Bioinformatics, Case Western Reserve University School of Medicine, Cleveland, Ohio, USA

**Keywords:** CTX-M, CTX-M-96, ESBL, KPC, KPC-2, MB_076, S02030, β-lactamases, boronate, carbapenemase

## Abstract

Design of novel β-lactamase inhibitors (BLIs) is one of the currently accepted strategies to combat the threat of cephalosporin and carbapenem resistance in Gram-negative bacteria. Boronic acid transition state inhibitors (BATSIs) are competitive, reversible BLIs that offer promise as novel therapeutic agents. In this study, the activities of two α-amido-β-triazolylethaneboronic acid transition state inhibitors (S02030 and MB_076) targeting representative KPC (KPC-2) and CTX-M (CTX-M-96, a CTX-M-15-type extended-spectrum β-lactamase [ESBL]) β-lactamases were evaluated. The 50% inhibitory concentrations (IC_50_s) for both inhibitors were measured in the nanomolar range (2 to 135 nM). For S02030, the *k*_2_/*K* for CTX-M-96 (24,000 M^−1^ s^−1^) was twice the reported value for KPC-2 (12,000 M^−1^ s^−1^); for MB_076, the *k*_2_/*K* values ranged from 1,200 M^−1^ s^−1^ (KPC-2) to 3,900 M^−1^ s^−1^ (CTX-M-96). Crystal structures of KPC-2 with MB_076 (1.38-Å resolution) and S02030 and the *in silico* models of CTX-M-96 with these two BATSIs show that interaction in the CTX-M-96–S02030 and CTX-M-96–MB_076 complexes were overall equivalent to that observed for the crystallographic structure of KPC-2–S02030 and KPC-2–MB_076. The tetrahedral interaction surrounding the boron atom from S02030 and MB_076 creates a favorable hydrogen bonding network with S70, S130, N132, N170, and S237. However, the changes from W105 in KPC-2 to Y105 in CTX-M-96 and the missing residue R220 in CTX-M-96 alter the arrangement of the inhibitors in the active site of CTX-M-96, partially explaining the difference in kinetic parameters. The novel BATSI scaffolds studied here advance our understanding of structure-activity relationships (SARs) and illustrate the importance of new approaches to β-lactamase inhibitor design.

## INTRODUCTION

The continuous and accelerated emergence of bacterial strains resistant to virtually all antibiotics is currently one of the most challenging public health issues. Due to the ubiquitous overuse of antibiotics, resistance mechanisms are continuously arising. Therefore, new treatment options for infections produced by multidrug-resistant (MDR) pathogens are required. In Gram-negative bacteria, production of β-lactamases is the most common mechanism of resistance to β-lactam agents (1, 2).

The increasing use of β-lactam antibiotics has favored the selection and proliferation of more than 7,700 class A β-lactamase variants belonging to the four Ambler’s classes (http://www.lahey.org/studies and http://bldb.eu) (3). The CTX-M enzymes are the most prevalent and relevant group of extended-spectrum β-lactamases (ESBLs) among pathogens around the world (2, 4, 5). CTX-M β-lactamases are generally differentiated from other ESBLs, as they show high catalytic efficiency toward cefotaxime and ceftriaxone but for the most part spare ceftazidime (6). The KPC β-lactamases present a larger and more ominous global threat. The expansion of this carbapenemase has presented challenges in detection, antibiotic treatment, and infection control measures. To overcome the action of these pandemic resistance determinants (CTX-Ms and KPCs), attention is focused on developing novel β-lactamase inhibitors (BLIs) that effectively inactivate the β-lactamases, thus protecting the partner antibiotic and allowing it to target the penicillin-binding proteins.

**FIG 1:**
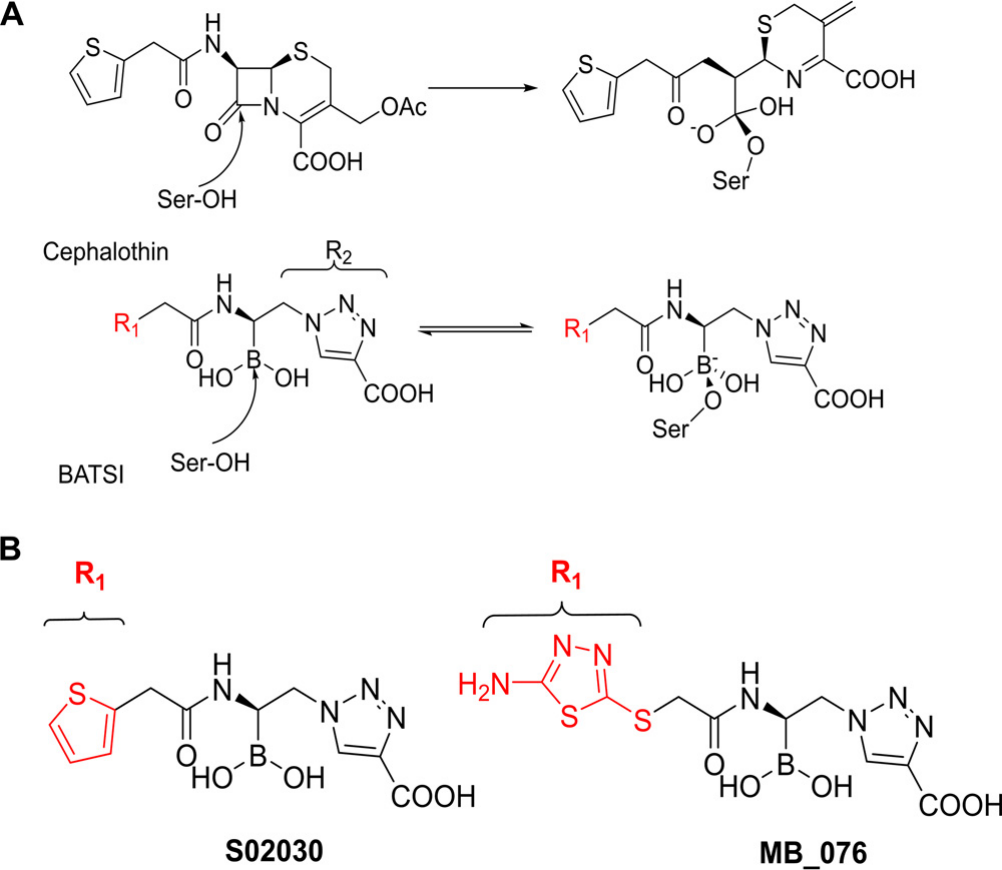
(A) Schematic representation of the BATSIs binding to the active site of a serine β-lactamase resembling the quaternary transitional state of the β-lactam hydrolysis reaction leading to inhibition. In the case of BATSIs, this is a reversible competitive process. (B) Chemical structures of the R1 moieties in the BATSIs S02030 and MB_076.

In the quest for new antibiotics, structure-based design is widely used to create new BLIs that mimic interactions observed between the target enzyme and its natural substrates (7). Attention to novel BLIs bearing an electrophilic center (phosphonates, aldehydes, trifluoromethylketones, and boronic acids) that can covalently modify the nucleophilic catalytic serine has advanced our thinking in the field (8). Boronic acid transition state inhibitors (BATSIs) have the potential to become clinically important BLIs, which would add to the success of FDA-approved vaborbactam, as they possess a boron atom acting as an electrophile that mimics the carbonyl carbon of the β-lactam ring and forms a tetrahedral adduct with the catalytic serine that closely resembles the transition state in the hydrolytic mechanism (Fig. 1A) (7, 9–11).

In this study, we further evaluated the inhibitory activities of two α-amido-β-azidoethane BATSI compounds (Fig. 1B); these compounds have a boron atom substituted with chemical groups that mimic those of the transition state of the natural substrate with the enzyme, i.e., the R1 amide side chain and the R2 heterocyclic ring bearing a carboxylate (Fig. 1A). This class of BATSIs has previously proved to be very active against KPC-2 and other ESBLs, in particular S02030 (12, 13). In addition, a CTX-M-15-type variant, CTX-M-96, which has 99% amino acid identity with CTX-M-15 (a single substitution, N89S, in the mature protein) was also examined (2). Our previous studies showed that S02030 was active against KPC carbapenemase (*k*_2_/*K* = [1.2 ± 0.2] × 10^4^ M^−1^ s^−1^ for KPC-2) and combined with cefepime restored susceptibility in Escherichia coli DH10B carrying *bla*_SHV_, *bla*_KPC-2_, and *bla*_KPC-3_ variants and against clinical strains of Klebsiella pneumoniae and E. coli possessing various class A β-lactamases (12). MB_076 was designed to improve oxidation stability and hydrophobic interactions in the active site of serine class A β-lactamases, replacing the thiophene ring of S02030 with an amino-thiadiazole ring. Here, we explored the interactions in structural complexes through X-ray crystallography (KPC-2 in complex with MB_076 compared to a previous structure of KPC-2 with S02030; PDB ID 5EEC) and *in silico* modeling (CTX-M-96 enzyme in complex with S02030 and MB_076). As a result of these efforts, we advanced our understanding of the structure-activity relationship (SAR) of S02030 and the amino-thiadiazole derivative, MB_076 (Fig. 1B). Attention is focused upon the molecular interactions and ability of these inhibitors to inactivate two widespread resistance determinants and serve as candidates for further development.

In a separate paper, and from previous results, we demonstrated that S02030 and MB_076 restore cefepime activity against a pan-drug-resistant KPC-producing K. pneumoniae clinical isolate. The BATSIs do not have antimicrobial activity alone, but when paired with cefepime, they demonstrate antibacterial activity comparable to that of ceftazidime-avibactam against infections caused by class A carbapenemase-producing *Enterobacterales*, particularly KPC (our unpublished data).

## RESULTS AND DISCUSSION

### Chemical structures of MB_076 and S02030.

The chemical structures of MB_076 and S02030 are shown in Fig. 1B. S02030 was synthesized as previously described (14). MB_076 syntheses will be presented in a separate publication (our unpublished data).

### Kinetics.

Using nitrocefin as an indicator substrate and 5 min incubation before steady-state velocities were determined, 50% inhibitory concentrations (IC_50_s) in the nanomolar range for KPC-2 and CTX-M-96 β-lactamases were measured, and both inhibitors bound CTX-M-96 with greater affinity than KPC-2 (Table 1). The IC_50_ was approximatively 40-fold lower for CTX-M-96 than KPC-2 for both inhibitors. The IC_50_ of S02030 is 2 nM against CTX-M-96 and 80 nM against KPC-2 (12). MB_076 has an IC_50_ of 4 nM against CTX-M-96 versus 135 nM for KPC-2. The on rates (*k*_2_/*K*) for S02030 and MB_076 versus CTX-M-96 were 24,000 M^−1^ s^−1^ and 3,900 M^−1^ s^−1^, respectively, and those for KPC-2 were 12,000 M^−1^ s^−1^ and 1,200 M^−1^ s^−1^ respectively (Table 1). These values indicate that S02030 possesses a more rapid on rate for both the CTX-M variant and KPC-2 enzyme (6-fold higher for CTX-M-96 and 10-fold higher for KPC-2 compared to MB_076). Compared to other BLIs, the *k*_2_/*K* obtained for both BATSIs (especially S02030) remained within the range of the inactivation efficiency value (*k*_inact_/*K*_I_) of clavulanic acid previously reported for CTX-M-96 (45,000 M^−1^ s^−1^) (15), and also for other ESBLs and class A carbapenemases (e.g., KPC-2, CTX-M-9, PER-2, etc.), whose values range between 2 × 10^3^ and 6 × 10^4^ M^−1^ s^−1^ (12, 15). The *k*_2_/*K* values for both BATSIs against CTX-M-96 were lower than those of avibactam (16). The off-rate (*k*_off_) values were 0.00075 s^−1^ for S02030 and 0.00062 s^−1^ for MB_076, which were similar to those of KPC-2 (0.00046 s^−1^) (12), suggesting similar recovery rates in both enzymes for these BATSIs (Table 1). Kinetic plots for CTX-M-96–S02030, CTX-M-96–MB_076, and KPC-2–MB_076 are shown in Fig. S1 in the supplemental material.

**TABLE 1:** Comparative inhibition parameters of CTX-M-96 and KPC-2 class A β-lactamases against S02030 and MB_076

β-Lactamase	S02030			MB_076		
	IC_50_ (μM)	*k*_2_/*K* (M^−1^ s^−1^)	*K*_off_ (s^−1^)	IC_50_ (μM)	*k*_2_/*K* (M^−1^ s^−1^)	*K*_off_ (s^−1^)
CTX-M-96	0.002 ± 0.0002	24,000 ± 900	(0.75 ± 0.03) × 10^−3^	0.004 ± 0.0005	3,900 ± 500	(0.62 ± 0.05) × 10^−3^
KPC-2^*a*^	0.08 ± 0.002	12,000 ± 2,000	(0.46 ± 0.02) × 10^−3^	0.135 ± 0.02	1,200 ± 300	(1 ± 0.2) × 10^−3^

a Data for KPC-2 versus S02030 were published previously (12).

### Crystallography and *in silico* modeling.

**FIG 2:**
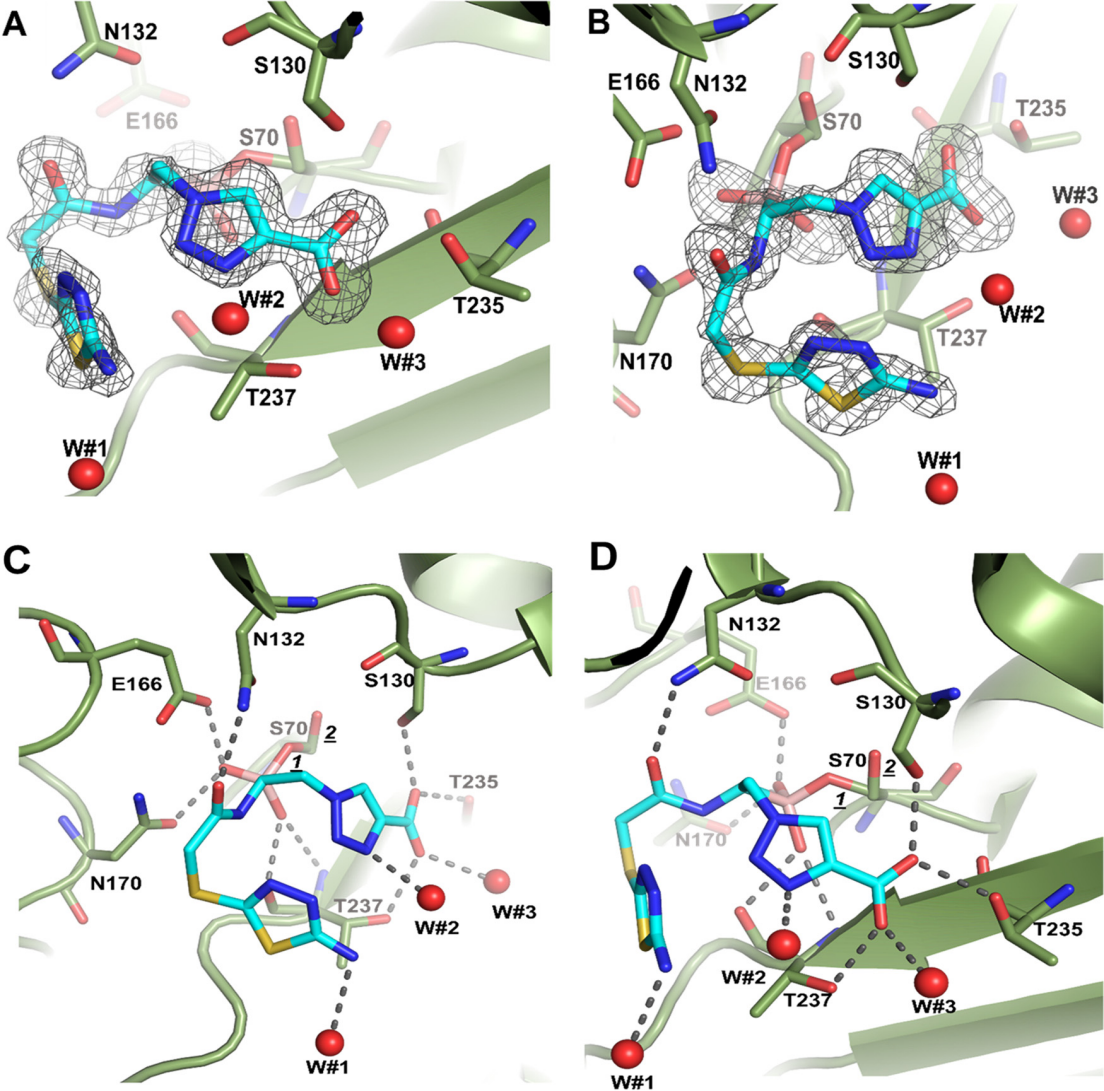
Structure and the electron density of MB_076 bound covalently in the active site of KPC-2 β-lactamase. Unbiased omit F_o_-F_c_ difference density (A) was obtained after removing MB_076 from the model and subsequently performing 10 cycles of crystallographic refinement prior to the map calculation. KPC-2 is shown as a green backbone trace with green carbon atoms. MB_076 is shown in a stick model with cyan carbon atoms. The difference density is contoured at the 2.75 σ level. (C) Close-up view of MB_076 in the KPC-2 active site. Hydrogen bonds are depicted as dashed lines. Crystallographic water molecules interacting with MB_076 are shown as red spheres (W#1, W#2, and W#3). The alternative conformations of S70, with the hydroxyl pointing in a slightly different orientation, are labeled *1* (0.8 occupancy) and *2* (0.2 occupancy). (B, D) Same as panels A and C, but the view is rotated about 90°.

**(i) Crystal structure complex of KPC-2 and MB_076.** A crystal structure of KPC-2 with MB_076 bound was solved at 1.38 Å resolution. The final model refined to an *R*_work_ value of 14.8% and an *R*_free_ value of 17.0% (additional data and refinement statistics are listed in Table 2). The unbiased omit map contoured at 2.5 σ revealed density for MB_076 being covalently attached to the Oγ atom of the catalytic S70 residue. MB_076 was refined to an occupancy of 0.8 (Fig. 2A and B). The electron density is present for the triazole ring, the amino-thiadiazole ring, the two boronic acid hydroxyl moieties, and the carboxyl group (Fig. 2A). The two boronic acid hydroxyls of MB_076 form stabilizing interactions and occupy the oxyanion hole (formed by backbone N:H exchangers [NHEs] of S70 and T237) and the deacylation water pocket (formed by E166 and N170) of KPC-2. The S70 has a minor second conformation (0.2 occupancy, labeled “*2*” in Fig. 2) that is not bonded to MB_076 and has the hydroxyl pointing in a slightly different orientation (Fig. 2C and D).

**TABLE 2:** Data collection and refinement statistics

Data collection parameter	Value for KPC-2 with MB_076
Wavelength (Å)	0.97933
Resolution range	28.02–1.38 (1.43–1.38)
Space group	P 2_1_ 2_1_ 2_1_
Unit cell dimensions (Å)	51.72, 66.67, 73.14; 90, 90, 90
Unique reflections	52,526 (3,670)
Multiplicity	6.6 (6.1)
Completeness (%)	99.59 (96.33)
Mean *I*/sigma(*I*)	15.5 (2.2)
*R*_merge_	0.068 (0.683)
CC_1/2_	0.999 (0.767)
Refinement	
Resolution range	1.38–28.04
*R*_work_	0.148 (0.231)
*R*_free_	0.170 (0.253)
Ligand atoms	24
Solvent molecules	302
Protein residues	262
Ramachandran plot statistics	
Bond length (Å)	0.015
Bond angle (°)	1.88
Ramachandran favored (%)	98.46
Ramachandran allowed (%)	1.54
Rotamer outliers (%)	2.39

The carboxylate binding group of the MB_076 is situated in the β-lactam carboxyl binding pocket and forms hydrogen bonds in the active site with S130, T235, and T237. The carbonyl oxygen in MB_076 interacts and forms a hydrogen bond with N132 (Fig. 2C). The C-H of the triazole ring is polar and can form a 3.0-Å C-H—O hydrogen bond interaction with the hydroxyl of S130. In accordance with our goal (to improve hydrophobic interactions by its thiophene ring) the CH_2_-S-thiadiazole ring-containing portion of MB_076 engages in hydrophobic interactions with N170, the main chain atoms of C238, and T237. Furthermore, the amino-thiadiazole ring forms intramolecular edge-to-face π-π interactions with the triazolyl moiety of MB_076. The interaction of MB_076 binding also involves three water molecules (Fig. 2C). Furthermore, we noticed a difference in W105 side chain orientation in the complex compared to the KPC-2 apo structure (PDB ID 2OV5) (17). This flexibility seems to increase the size of the active site, which accommodates a better fit. The role and the importance of this residue (W105) were also explored by Papp-Wallace et al. (18). This interaction is discussed in detail below.

**FIG 3:**
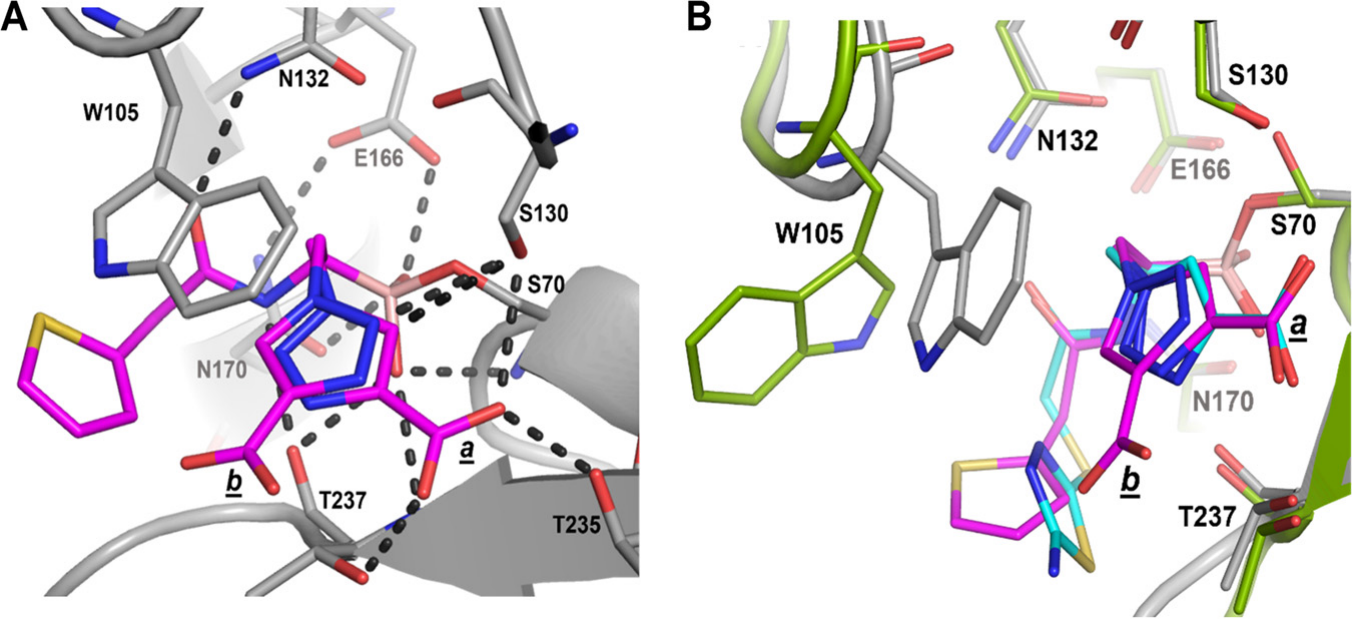
(A) Interactions of S02030 (magenta atom carbons) in the active site of class A KPC-2 β-lactamase (PDB ID 5EEC). Hydrogen bonds are depicted by dashed lines. The two conformations of the carboxyl-triazole are indicated by the labels *a* and *b*. (B) Superimposition of S02030 (magenta) bound to KPC-2 onto KPC-2 β-lactamase bound to MB_076 (cyan). The colors and representation of KPC-2 and its bound MB_076 are as in Fig. 2. KPC-2 bound to S02030 (PDB ID 5EEC) is shown as a gray backbone trace with gray carbon atoms; S02030 is depicted in stick format with magenta carbon atoms.

**(ii) Structural comparison of S02030 binding to KPC-2 β-lactamase.** Using the previously published structure of S02030 bound to KPC-2 (Fig. 3A) (PDB ID 5EEC) (19), we compared the modes of binding of S02030 and MB_076 to KPC-2 β-lactamase. MB_076 and S02030 are very similar in their chemical structure, with the only difference being that MB_076 has an *S*-thiadiazole ring on the side chain while S02030 has a thiophene ring (Fig. 1B).

Comparison of the KPC-2-complexed structures of MB_076 and S02030 reveals similarities and differences in binding mode and active site conformation (Fig. 3B). First, both compounds make a covalent bond with catalytic serine via the boron atom and have one of their boronic acid oxygens positioned in the oxyanion hole. Second, S02030 has two conformations for the carboxyl-triazole moiety (conformations labeled *a* and *b* in Fig. 3), while MB_076 has only one. One of the conformations (conformation *a*) overlaps the carboxyl-triazole moiety of MB_076, and both have interactions with the same residues (T237, T235, and S130), while the second conformation (conformation b) has a flipped triazole ring, and its carboxyl-triazole moiety is pointing downward (Fig. 3B).

Third, both compounds have a 1,2,3-triazole ring, which has the ability to participate in a unique hydrogen bond where the C-H functions as a hydrogen bond donor because of the polarity of the triazole ring due to the 3 nitrogen atoms on the other side of the ring, as was previously described by Kumar and Pandey (20), by Hua and Flood (21), and in our work (19). As a result of having the triazole ring, both compounds can form a special C-H—O hydrogen bond with S130. In S02030, the C-5 ring carbon of the triazole moiety of conformation b is at a 2.9-Å distance from the Oγ of S130 (Fig. 3) suggesting a C-H—O hydrogen-bonding interaction. For MB_076, the C-H of the triazole ring is at a 3.0-Å distance from the Oγ of S130, also suggesting a C-H—O hydrogen bond interaction (Fig. 2C and 3A).

Furthermore, unlike in MB_076, the thiophene ring of S02030 is not observed in edge-to-face π-π interactions with the triazolyl moiety; this could be due to the shorter linker in S02030. The shorter linker in S02030 has one less rotatable bond than MB_076, which would lead to less entropy loss upon binding for S02030 than MB_076. Also, unlike MB_076, the amide nitrogen of S02030 engages in an additional hydrogen bonding interaction with the backbone oxygen of T237 (Fig. 3A).

Finally, the binding of S02030 and MB_076 in the active site of KPC-2 forced the W105 side chain to accommodate a different orientation in each complex (Fig. 3). Additionally, S02030 has π stacking interactions with the W105 indole ring, which improved its binding affinity (12). Overall, these differences observed in MB_076 and S02030 binding to KPC-2 could contribute to their slight differences in affinity (Table 1).

**FIG 4:**
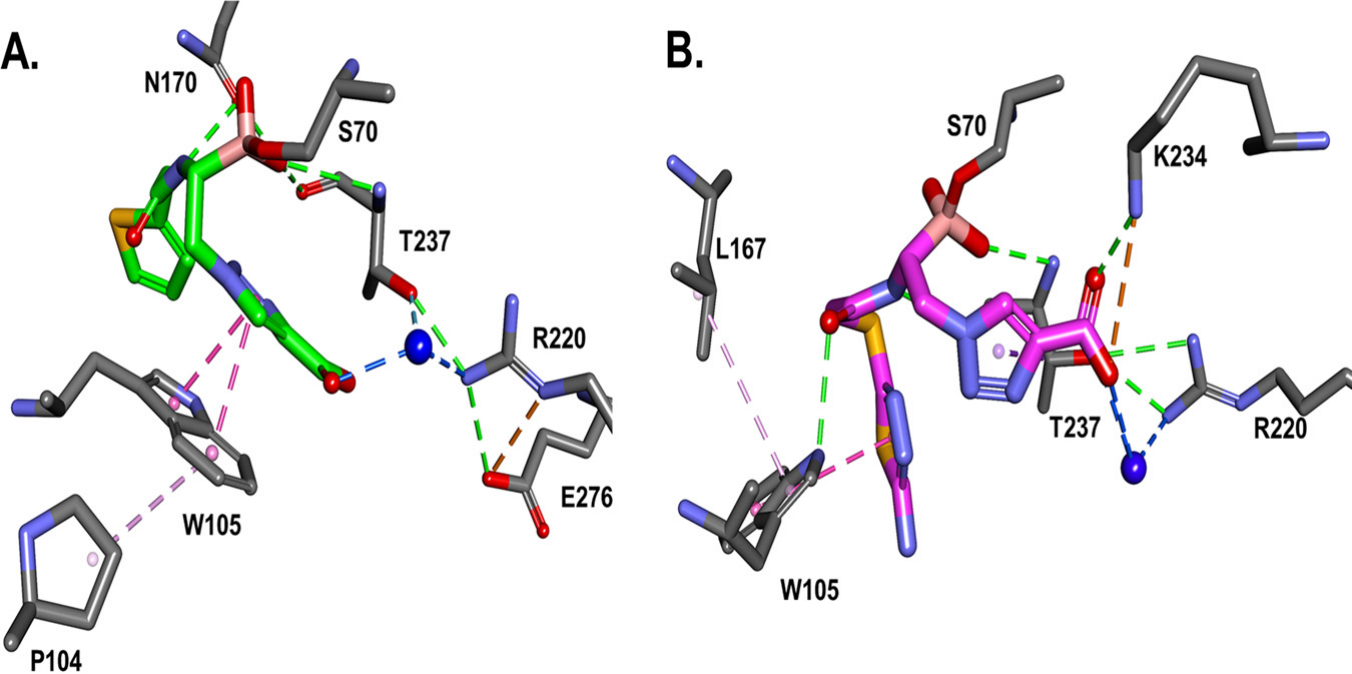
The crystal structures, followed by molecular dynamic simulation of the KPC-2–S02030 (A) and KPC-2–MB_076 (B) complexes, show slightly different binding patterns. The carboxyl group of MB_076 engages in more direct interactions with R220, T237, and K234 compared with water-mediated interaction of S02030 with R220. However, W105 engages in stacking interactions with the S02030 thiophene ring and P104, versus edge interactions with MB_076 and hydrophobic interactions with L167.

To assess the flexibility of the conformation of W105 in relation to the BATSI, the crystal structure analysis was followed by 500-ps molecular dynamic simulation (MDS) of the KPC-2–S02030 (Fig. 4A) and KPC-2–MB_076 (Fig. 4B) complexes. During MDS, the compounds show a different binding pattern. The carboxyl group of MB_076 engages in more direct interactions with R220, T237, and K234 than during the water-mediated interaction of S02030 with R220.

**FIG 5:**
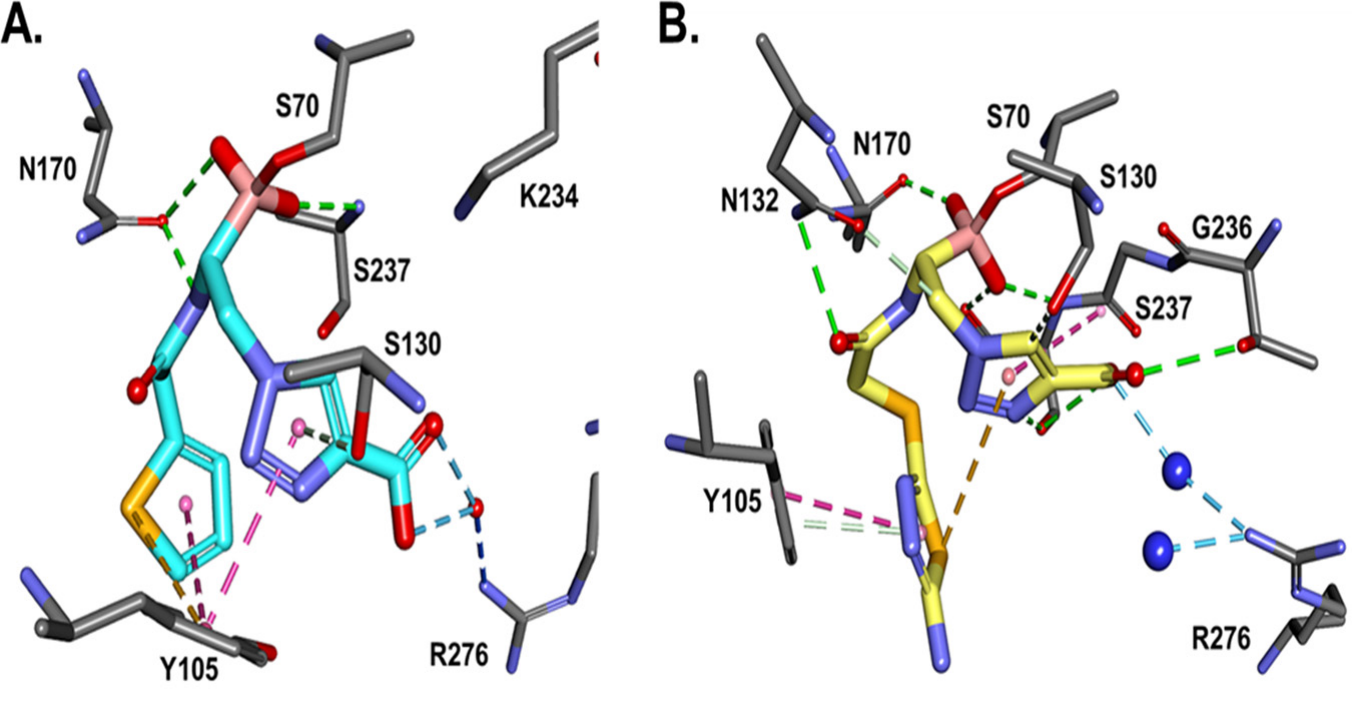
*In silico* model of S02030 (A) and MB_076 (B) in the active site of CTX-M-96. Molecular interactions of S02030 and MB_076 R1 groups with Y105 are similar: S02030 thiophene and MB_076 S-thiadiazole rings engaging in π stacking interactions. The main difference is in R2 carboxyl binding; both BATSIs interact with R276 through a water molecule, but MB_076 interacts directly with G236 and T237 as well. Favorable hydrogen bonds are depicted as green slashed lines, hydrophobic ones are magenta, and water H bonds are blue.

**FIG 6:**
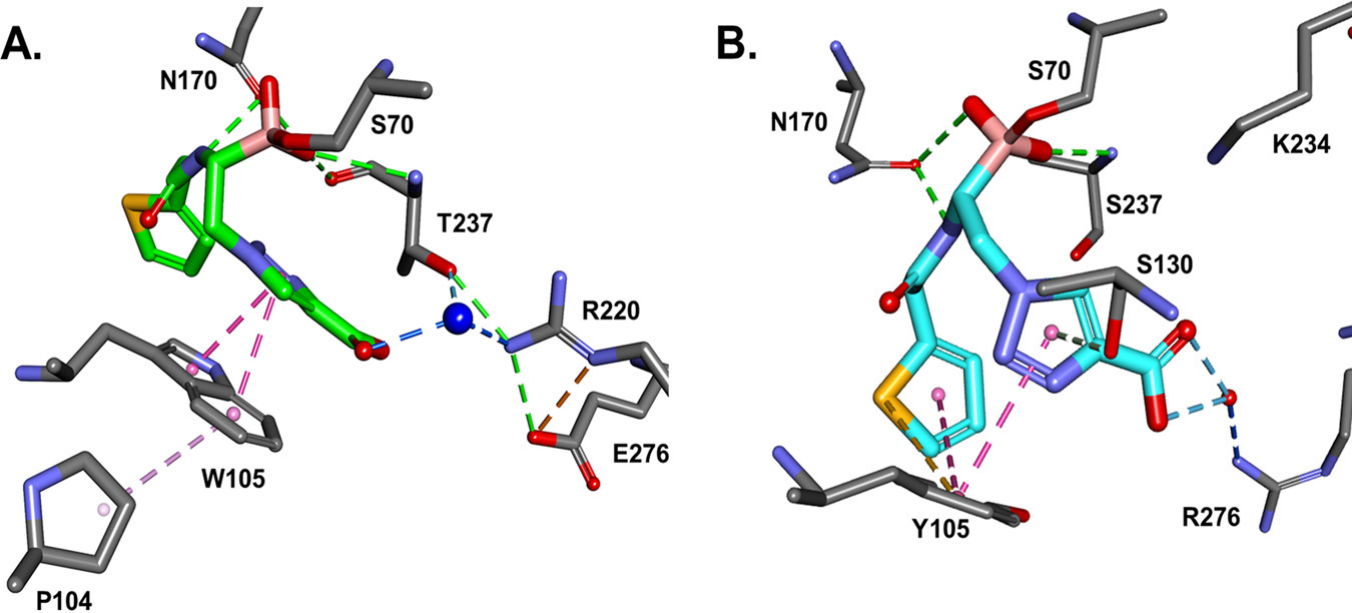
When the S02030 binding with KPC-2 (A) is compared with the S02030 binding with CTX-M-96 (B), the main difference is the positioning of the tetrahedral geometry around the boron atom. The hydrophobic interactions with the S02030 thiophene ring are present for both KPC-2 (W105), and CTX-M-96 (Y105). However, W105 in KPC-2 seems to be engaged in hydrophobic interactions with P104 as well, which may contribute to more rigidity in KPC-2–S02030 versus CTX-M–S02030, resulting in a slightly higher affinity of S02030 for CTX-M-96.

**(iii) Molecular modeling of S02030 and MB_076 into the CTX-M-96 active site.** The energy-minimized *in silico* model of the S02030–CTX-M-96 complex (Fig. 5A) predicts interactions equivalent to that observed for the crystallographic structure of KPC-2–S02030 (Fig. 3A), with favorable hydrogen bond interactions that may involve S70, S130, N132, N170, and S237. A tetrahedral geometry around the boron atom of S02030 is observed; the boronate group forms covalent bonds with the active site S70 and creates hydrogen bonds with the backbone nitrogen atoms of S70 and S237 in the oxyanion hole and with N170. Compared to the complex with KPC-2 (Fig. 6A), the carboxy-triazole side chain of S02030 (through its terminal *m*-carboxylate group) is likely involved in a salt bridge that eventually interacts with R276 instead of R220 as in KPC-2, although crystallographic structures will need to confirm this interaction. In addition, the modeling indicated that π-stacking interaction between the triazole *R2* chain of S02030 and Y105 in CTX-M-96 likely occurs as well (Fig. 6B) (~3.6-Å distance between the S02030 triazole ring and W105 in KPC-2).

To compare, *in silico* modeling of the complex CTX-M-96–MB_076 shows that the MB_076 could fit in a manner similar to that of S02030 in the active site, with equivalent interactions with the main residues S70, S237 (in the oxyanion hole), and N170 but also different interactions (Fig. 5B). The most remarkable difference seems to be the interaction of the carboxy-triazole ring of MB_076 with T216 and G236 through hydrogen bonds. The interaction with R276 seems to be mediated by a water molecule for both boronic acid inhibitors and a direct H bond with G236 for MB_076. Also, a van der Waals interaction between the aminothiadiazole moiety of MB_076 (via its primary amino group) and the S237 seems to occur. Whether these structural differences have an impact on the observed inhibition kinetic parameters remains to be demonstrated through experimentally determined crystallographic structures of the MB_076 compound with CTX-M-96.

**FIG 7:**
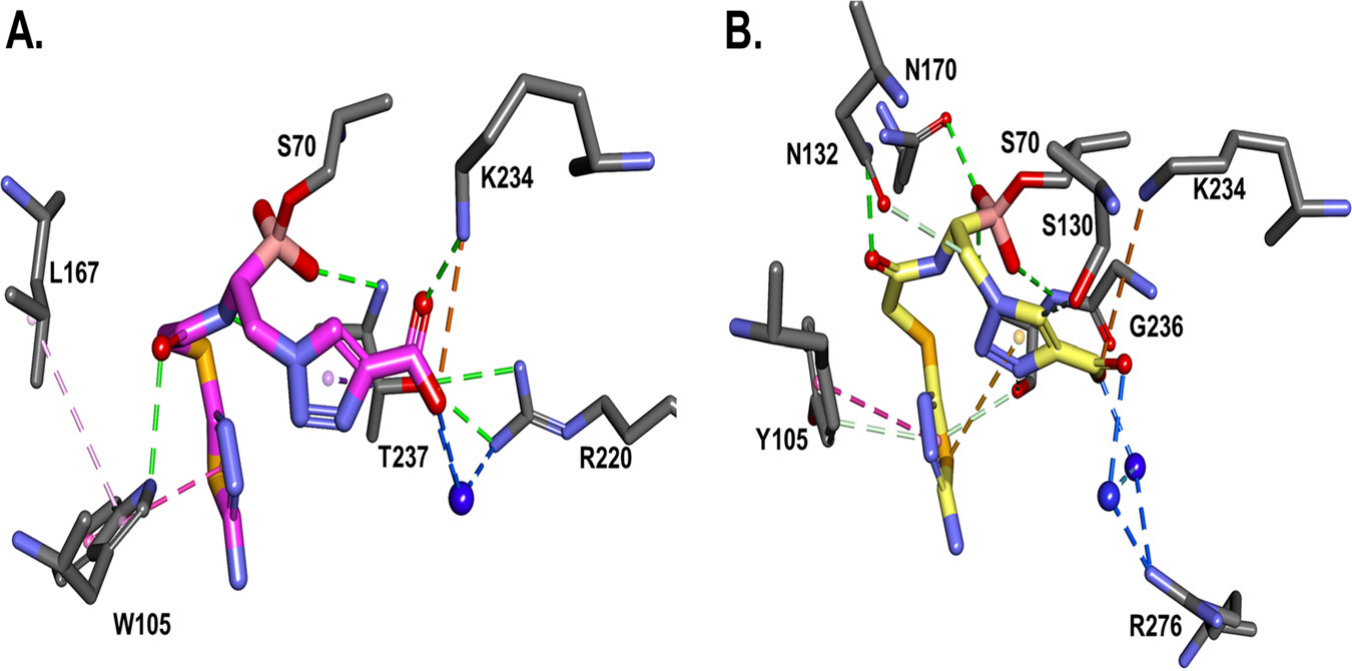
When the MB_076 binding with KPC-2 (A) is compared with the MB_076 binding with CTX-M-96 (B) after 500 ps molecular modeling simulation, the main difference is H-bond interactions of the boronic tetrahedral geometry, with more H bonds present in CTX-M-96 than in KPC-2. The notable interaction is the direct ionic bond of the carboxyl group with K234 residue. The hydrophobic interactions with MB_076 S-thiadiazole ring are present for both KPC-2 (W105) and CTX-M-96 (Y105). However, as for the S02030 complex, W105 in KPC-2 seems to be engaged in hydrophobic interactions with L167 for this complex, resulting in a slightly higher affinity of MB_076 for CTX-M-96.

When MB_076 binding with KPC-2 is compared to binding of MB_076 with CTX-M-96, the main difference is the interaction network displayed by the tetrahedral boron moiety (Fig. 7). The hydrophobic interactions with the MB_076 S-thiadiazole ring are present for both KPC-2 (W105) and CTX-M-96 (Y105). However, W105 present in KPC-2 seems to be engaged in hydrophobic interactions with P104 as well, which may contribute to more rigidity in the KPC-2–MB_076 complex versus the CTX-M–MB_076 complex, resulting in a slightly higher affinity of MB_076 for CTX-M-96. Similarly, when MB_076 binding with KPC-2 is compared with MB_076 binding with CTX-M-96 after molecular modeling simulation, the main difference is H-bond interactions of the boronic tetrahedral moiety, with more H bonds present in CTX-M-96 than in KPC-2. KPC-2 seems to be restrained in hydrophobic interactions with L167 for this complex, resulting in a slightly higher affinity of MB_076 for CTX-M-96 enzyme.

In summary, we provide the biochemical and structural basis for the evaluation of MB_076, a derivative of S02030 with improved solubility (1 M stock solution in dimethyl sulfoxide [DMSO], versus 250 mM stock solution for S02030). Studies evaluating the microbiological and *in vivo* efficacy of MB_076 are presented in a separate analysis. Our current data, as well as the ongoing efforts to obtain crystallographic structures of other covalent complexes of other class A β-lactamases (particularly CTX-Ms and KPCs) with both BATSIs, will provide further insights into the recognition of these non-β-lactam compounds by class A enzymes and will serve as a starting point for the optimization of these boronic acid derivatives, as well as other potentially unexplored chemical compounds, as promising alternatives to counteract the antimicrobial resistance problem.

## MATERIALS AND METHODS

### Protein purification.

The β-lactamase KPC-2 was expressed and purified as previously described (22). The KPC-2 pET30a plasmid was transformed into E. coli BL21 Star competent cells. Terrific broth medium (TB) with kanamycin 50 μg/mL was used for the large (3- to 4-L) cultures; protein expression was induced by adding a final concentration of 50 μM isopropyl-β-d-thiogalactopyranoside (IPTG) when the optical density at 600 nm (OD_600_) reached 0.7. The cultures were shaken continuously for 18 h at 18°C as described previously (17, 22). Subsequently, the cells were collected by centrifugation and were suspended in lysis buffer (20 mM Tris [pH 8], 300 mM NaCl, a complete EDTA-free protease inhibitor tablet [Sigma-Aldrich], 1,000 U of Benzonase [ACROBiosystems], and 3 mM MgCl_2_). Cells were lysed twice using an Avestin Emulsiflex B-15 apparatus at 4°C; the cell suspension was then cleared by centrifugation at 15,000 rpm for 35 min. Polyethyleneimine (PEI; 0.1%) from a 6.5% (vol/vol) stock was added to the supernatant to precipitate contaminating DNA and RNA and was incubated with shaking at 4°C for 20 min followed by centrifugation at 8,000 rpm for 15 min. The supernatant was incubated with washed phenyl-boronic acid agarose beads (Sigma-Aldrich) for 16 h at 4°C and then washed with 20 mM Tris (pH 7.9), 150 mM NaCl_2_ buffer. The KPC-2 protein was eluted with 0.85 M Tris (pH 7.9), 0.2 M sorbitol, and 0.2 M NaCl. The protein was further purified using Superdex 75 size exclusion column chromatography with 10 mM Tris (pH 8), 60 mM NaCl, and 10 mM sorbitol. Finally, the peak fractions containing purified KPC-2 were collected and concentrated.

CTX-M-96 was purified to homogeneity as described previously (15). The plasmid pET96, a pET28 derivative containing the cloned *bla*_CTX-M-96_ gene, obtained in a previous study (2), was introduced by transformation in competent E. coli BL21(DE3) cells upon selection in lysogeny broth (LB) agar plates with kanamycin (30 μg/mL). An overnight culture of a BL21(DE3)/pET96 clone at 37°C in LB broth containing 30 μg/mL kanamycin was diluted 1/20 in the same culture medium and grown at 37°C to an OD_600_ of 0.6, and 1 mM IPTG (isopropyl-β-d-thiogalactopyranoside) was added to induce β-lactamase expression. After 20 h of incubation at 18°C, crude extracts were obtained by sonication, and clear supernatants containing CTX-M-96 were dialyzed against buffer A (50 mM Tris, 200 mM NaCl; pH 8) and purified by affinity chromatography using HisTrap columns (GE Healthcare, USA) by elution with linear gradient of buffer B (buffer A with 500 mM imidazole; pH 8.0). Highly pure CTX-M-96 was digested with thrombin for His tag removal before kinetic assays.

### Kinetics.

The inhibition by BATSIs is assumed to follow a reversible mechanism according to the following scheme:

(1) $$\text{E}+\text{I}↔\text{E}:\text{I}↔\text{E}-{\text{I}}^{\text{*}}↔\text{E}-{\text{I}}^{\text{**}}$$

where E represents the enzyme, I the BATSI, E:I the Michaelis complex, E-I* the enzyme-inhibitor complex as the acylation transition state analog, and E-I** the deacylation transition state intermediate, according to previous models (15). The half-maximal inhibitory concentration (IC_50_) was measured after a 5-min preincubation of the enzyme and each BATSI at increasing concentrations, with 100 μM nitrocefin as the reporter substrate, as previously described (12, 13).

To determine the on rate (*k*_2_/*K*), progress curves were obtained by incubating KPC-2 or CTX-M-96 with increasing concentrations of BATSIs and 100 μM nitrocefin as the reporter substrate. Progress curves were fitted to equation 2 to obtain *k*_obs_ values, by nonlinear least-squares fitting of the data, and *k*_2_/*K* was determined from equation 3.

(2) $$A=\;v_{f\;}\text{ * }t+\left(v_{0}-v_{\text{f}}\right)\times (1-\;e^{-k_{\mathrm{obs}}t})/k_{\text{observed}}+A_{0}$$

(3) $$k_{\text{observed}}=k_{-2}+(k_{2}/K)\times ([I]/(1+([S]/K_{m\text{ nitrocefin}})))$$

In equation 2, *A* is absorbance at 482 nm, *v*_f_ is final velocity, *t* is time, *v*_0_ is initial velocity, and *A*_0_ is initial absorbance at 482 nm. In equation 3, [*I*] is the concentration of BATSI and [*S*] is the concentration of nitrocefin. The data were plotted as *k_obs_* versus [BATSI], and the *k*_2_/*K* value was obtained by correcting the value for the slope of the line for the concentration and affinity of nitrocefin (Equation 4).

(4) $$k_{2}/K_{\text{corrected}}=k_{2}/K_{\text{observed}}\times (([S]/K_{m\text{ nitrocefin}})+1)$$

The *k*_off_ value was determined by mixing KPC-2 or CTX-M-96 with 100 μM nitrocefin after preincubating 5 min with a saturating amount of each BATSI, and progress curves were fitted to a single exponential decay equation as previously described (12–14).

### Crystallization and data collection.

Crystals of KPC-2 were grown using the sitting drop method. The protein was concentrated to 17 mg/mL in 10 mM Tris (pH 8), 60 mM NaCl, 10 mM sorbitol; 1 μL of protein was mixed with 0.5 μL of reservoir containing 100 mM citric acid (pH 5.5), 100 mM KSCN, 30% polyethylene glycol (PEG) 6000, and 10 mM CdCl_2_.

The KPC-2 crystals were soaked for 48 h in MB_076 and frozen in liquid nitrogen for data collection. The crystal structures were solved by molecular replacement using the program PHASER with chain A of the KPC-2 structure (PDB ID 2OV5) (17) as the initial search model. The crystallographic refinement was carried out by applying several rounds of REFMAC (23) and manual model building with COOT (19, 24) (Table 2). The MB_076 KPC-2 complex was refined to 1.38 Å resolution.

### Molecular modeling.

*In silico* modeling of the CTX-M-15-type β-lactamase in complex with both BATSIs was obtained using the X-ray structure of CTX-M-96 (PDB ID 3ZNY) (13) as a template. The spatial coordinates of S02030 and MB_076 were obtained from the X-ray structures of KPC-2 in complex with S02030 (PDB ID 5EEC) (22) and MB_076 (PDB ID 7UTB), respectively. The MB_076 compound was built using Discovery Studio 2020 Client (25) molecular modeling software and energetically minimized using a CHARMm algorithm. The covalently linked structures (CTX-M–S02030 and CTX-M–MB_076) were energy minimized using a conjugate gradient method, with a root mean square (RMS) gradient of 0.001 kcal/(mol × Å). The GBSW (generalized born with a simple switching) solvation model was used, and long-range electrostatics were treated using a particle mesh Ewald method with periodic boundary condition. The SHAKE algorithm was applied. In order to assess the stability of the complexes, MDS was run for crystallographic (KPC-2 with S02030 and MB_076) and model-generated (CTX-M-96 with S02030 and MB_0760) structures. The generated trajectories (conformations) during the 500-ps MDS were analyzed for energetic stability.

### Data availability.

The structure factors and coordinates have been deposited in the Protein Data Bank (7UTB).
