# Supplementary figures and images for: Boronic Acid Transition State Inhibitors as Potent Inactivators of KPC and CTX-M β-Lactamases: Biochemical and Structural Analyses
Source: Antimicrob Agents Chemother. 2023 Jan 5;67(1):e00930-22. doi: 10.1128/aac.00930-22 (PMC9872677; doi:10.1128/aac.00930-22)

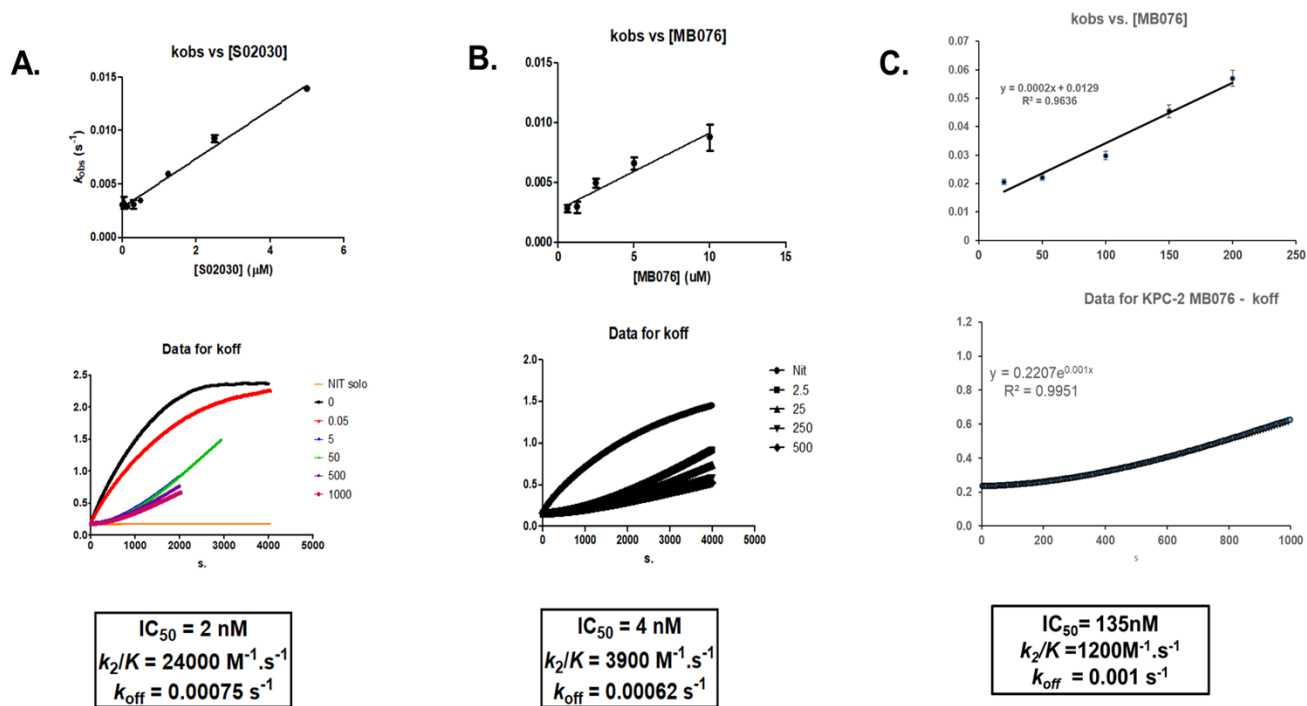

**Figure S1.** (A) CTX-M-96 S02030, (B) CTX-M-96 MB\_076 and (C) KPC-2 and MB\_076 kinetics.

Supplement: Supplemental file 1 — Fig. S1. Download aac.00930-22-s0001.pdf, PDF file, 0.2 MB [file aac.00930-22-s0001.pdf]
